# Assessing lactococcal and enterococcal strains derived from traditionally fermented kefir and nabeez for their prospects as probiotics

**DOI:** 10.1099/mic.0.001616

**Published:** 2025-10-15

**Authors:** Ghazal Aziz, Arsalan Zaidi

**Affiliations:** 1 National Probiotic Laboratory, National Institute for Biotechnology and Genetic Engineering College (NIBGE-C)-PIEAS, Faisalabad 38000 (Punjab), Pakistan

**Keywords:** antagonism, *Enterococcus*, probiotics, safety, simulated gastrointestinal fluids, traditional fermented beverages

## Abstract

Consumers' healthy lifestyle practices have heightened the appeal of minimally processed foods, especially the fermented kind. Kefir and nabeez with numerous benefits are world-famous beverages. This study aimed to explore the enterococcal and lactococcal probiotic strains associated with these beverages. Artisanal recipes were used to make kefir and nabeez, and bacteria were isolated using classical culturing techniques. The isolates were screened based on antimicrobial potential, safety and probiotic attributes. The bacterial isolates obtained from three fermented beverages, milk kefir, water kefir and nabeez, were assessed for safety concerns, and those deemed safe were tested for antagonistic potential. Strains of *Enterococcus durans* (NPL1395_MK_, NPL1396_MK_ and NPL1480_MK_), *Enterococcus faecium* (NPL1390_MK_, NPL1420_WK_ and NPL1427_Nb_) and *Lactococcus lactis* (NPL1426_Nb_, NPL1428_Nb_ and NPL1436_Nb_) demonstrated interesting antimicrobial characteristics against food-borne pathogens. Strains from milk kefir and nabeez could tolerate strong acidic and bile stress. All strains were susceptible to lysozyme and phenol at the concentrations tested but demonstrated significant antioxidant potentials, exopolysaccharide production and bile salt hydrolase activities. Cholesterol assimilation was most significant in milk kefir and nabeez strains, which also had good adherence and biofilm formation. Statistical analysis of performance data using the principal component analysis identified *L. lactis* strain NPL1428_Nb_ as the best. It exhibited good potential to persist in the human gut based on its ability to tolerate *in vitro* mixtures simulating the gastrointestinal tract digestive fluids, using the static digestion model. Therefore, strain NPL1428_Nb_ of traditional fermented beverage provenance has good prospects for use in probiotic product development.

## Introduction

Only lately has the craft of fermentation – which primarily evolved to preserve food – been recognized for its profound health benefits, which can be linked to the numerous microbiota–substrate pairings that have produced a wide range of fermented foods [1]. Several traditionally fermented foods with socio-religious significance, such as kefir and nabeez, are valued highly in contemporary fermented food culture. Kefir holds unique appeal, having acquired its name from the Turkish word ‘keyif’ which means ‘good feeling’ or ‘pleasure’ [2,3]. It stands apart from other fermented products because of its unique starter culture [4] and aroma [5]. Unlike conventional fermentation, only starter grains from a prior fermented product (pitching) can initiate kefir production, and back-slopping is of little use [6,8]. The kefir grain is a matrix of polysaccharide (kefiran) and protein carrying a complex symbiotic blend of bacteria and yeast [2,5]. Where it is sourced from is particularly essential because the microbiological composition of kefir depends on agronomic practices and geographical region [9]. Kefir has health-promoting effects because of its anti-cancerous, anti-oxidant, anti-bacterial and anti-allergic effects [9,11]. Its use following antibiotic treatment is particularly beneficial [12], and it has been widely lauded as a ‘superfood’ on social media [5].

Kefir can also be a part of vegan diets as ‘water kefir’ (also ‘acquakefir’, ‘sugary kefir’ and ‘tibico’), imparting health benefits comparable to its dairy counterpart [13]. They differ from milk kefir grain in their polysaccharide content, symbiotic associations, microbial species, the end product of fermentation [8] and catering to more diverse consumer categories like vegans and lactose intolerants [14]. Water kefir’s ability to exert antioxidant, anti-inflammatory, anti-cancerous, anti-microbial and immuno-modulatory effects has been documented in several clinical and preclinical studies [15,19]. The microbes in water kefir are metabolically versatile, thriving on various substrates, and several have been identified as probiotic candidates [8,20].

Nabeez, a popular Middle Eastern date-based beverage, has medicinal value [21]. It can be made by fermenting sweet, aqueous solutions of grains and fruits, dates being the preferred choice [22]. The date’s nutritional content endows nabeez water with natural antioxidants which help combat various disease conditions [23]. This high-energy drink helps detoxify the body, mitigates stomach acidity, maintains body hydration and increases human milk production and infant weight [24,26].

Because of their high microbiological loads, these fermented products have a limited shelf life. Standardizing these products using specific microbes in singly or in consortia is a promising method for preserving their desirable properties [2]. Understanding the microbial communities autochthonous to the fermented food is essential to help improve its quality and safety [27]. Two lactic acid bacteria (LAB) genera that stand out in this context are the *Lactococcus* and *Enterococcus*. The scientific community has often questioned the beneficial role of enterococci [28]. Their potential as probiotics is hampered by the frequent presence of resistance or virulence factors in them, despite the fact that they are gut commensals with good gut colonization ability [29]. However, thorough characterization of candidate strains can help distinguish commensals from clinical ones [30]. *Lactococcus lactis*, on the other hand, is a well-known LAB sp., which is frequently employed in feed fortification and fermentation, but occasionally, it too has been the subject of clinical controversy [26]. *L. lactis* strains in the past have typically passed the regulatory gauntlets of generally recognized as safe (GRAS) and qualified presumption of safety (QPS) certifications, but rising concerns of pathogenicity merit a re-examination of its innocuous status [26]. Because of safety concerns, *Enterococcus* and *Lactococcus* strains, especially those of traditional fermented beverage provenance, have quite often been bypassed as probiotic prospects.

Both of these understudied fermented beverages are associated with the mitigation of symptoms of multiple ailments and are believed to bear ‘beneficial bacteria’. Therefore, this study aims to isolate and characterize bacterial strains associated with traditional fermented beverages with the potential for use as probiotic organisms [1].

## Methods

### Preparing the fermented beverages

#### Ingredients and sourcing

Milk kefir and water kefir grains were purchased from an online ‘nature’s store’ (www.thenaturesstore.com) advertised as ‘kefir milk 100% active grains’ and ‘kefir water 100% active grains’, respectively. Both were labelled as non-genetically modified organisms and gluten-free. A commercial ultra-high temperature (UHT)-treated whole cream cow milk (5% fat, 7% protein and 5% carbohydrates), devoid of any chemical additives and preservatives, was used for making milk kefir grains. Nabeez was made using commercially available Ajwa dates (*Phoenix dactylifera* L.). Granulated brown sugar and sterile mineral water (pure life) were used to make water kefir and nabeez.

#### Recipe for nabeez

Nabeez was prepared following a traditional method mentioned earlier [21] with some modifications. The dates were washed and disinfected (5% sodium hypochlorite) for 10–20 min and thoroughly rinsed with sterile distilled water (DW) to remove residual disinfectant. Five grams of date flesh (seedless) were soaked in 100 ml of sterile pure water in a closed sterile glass container and allowed to stand for 18 h at room temperature (25 °C±1 °C). After fermentation, the dates were homogenized and filtered to get the nabeez drink.

#### Recipe for milk and water kefir

Kefir grains were revived and preserved as per the seller’s recommendation. Approximately 10 g was aseptically revived in 100 ml UHT-treated milk in the case of milk kefir and sterile sucrose water in the case of water kefir. Both were incubated at 25 °C for 24 h. Activation was done up to three successive passages before being finally stored at 4 °C.

Approximately 25 g of grains was aseptically measured and added to 250 ml commercial pasteurized milk in a sterile airtight glass jar to prepare the experimental milk kefir beverage. The grains were allowed to grow and ferment at 25 °C for 24 h [31]. Following fermentation, the grains were separated by filtration with a sterile sieve of 1–2 mm^2^ mesh size to get kefir as filtrate. The separated grains were collected aseptically and stored in sterilized UHT milk for up to a week.

Water kefir was prepared in filter-sterilized sucrose solution (10 g/100 ml) as cited [32] with some modifications. For beverage preparation, 80 g of water kefir grains was weighed and added to 250 ml of sucrose solution in a sterile glass jar (250 ml capacity) with the mouth covered with filter paper and incubated for 72 h in the dark at room temperature. After fermentation, the beverage was obtained after separating the grains by filtration with a sterile sieve of 1–2 mm^2^ mesh size. These grains were reused in a fresh sugar solution and fermented for a week.

The residual grains over the sieve were weighed gravimetrically to estimate kefir biomass [31]. Each beverage was prepared once in triplicate, and isolations were done after pooling the replicates.

### Bacterial isolation and preservation

Samples (1 ml) of each prepared beverage were 10-fold serially diluted in peptone water (0.1%, w/v), homogenized and spread plated (100 µl) on de Man, Rogosa and Sharpe (MRS) agar plates and incubated aerobically at 37 °C. Morphologically distinct and isolated colonies were picked, purified and preserved in 20% (v/v) glycerol at −40 °C.

### Preliminary bacteriological characterization

A total of 51 isolates were recovered from MRS agar plates which were observed for their colony characters, Gram-stained and tested for catalase activity. The bacterial growth kinetics of each isolate were noted by suspending overnight stock cultures in freshly made MRS broth (40 ml) in screw-capped Falcon tubes (50 ml) to a starting OD_600nm_ of 0.05. Cultures were maintained semi-aerobically at 37 °C without agitation for 24 h, and their growth was monitored spectrophotometrically by taking OD_600nm_ at 1 h intervals. Growth curves were plotted based on the increase in their biomasses (OD_600nm_) over time. The slope of the curve represents the exponential growth phase, and the steepest section of slope between two consecutive time points was used to calculate the maximum growth rate (µ/h), and the doubling times (T_d_) were also calculated accordingly as g=ln(2)/μ [33]. All subsequent assays were done using logarithmic phase cultures of the isolates in MRS broth.

### Screening the isolates for innocuousness

An array of *in vitro* tests was adapted to map out a safety profile of the strains used to make the test fermented products.

#### Haemolytic activity

Bacterial haemolytic activity was checked on Columbia sheep blood agar base supplemented with 5% (v/v) defibrinated human blood as recommended before [34]. After incubation, the plates were observed for the presence of a clear zone (*β*-haemolytic), greenish zone (*α*-haemolytic) or no zone (*γ*-haemolytic) around the bacterial colonies. A strain of *Staphylococcus aureus* (ATCC25923) was used as the positive control.

#### Gelatinase activity

Nutrient agar supplemented with 3% (w/v) gelatin [35] was used to assess gelatinase functionality. A loopful of log-phase culture was spotted on agar plates and incubated at 37 °C for 24–48 h. After incubation, the plates were refrigerated for 4 h and flooded with a saturated ammonium sulphate solution. A gelatin precipitation and transparent halos around the colonies indicated a functional gelatinase.

#### Mucin degradation

The ability to degrade mucin was checked as before [36]. Log-phase cultures of the isolates were spotted (~2 µl) on MRS agar reconstituted with mucin (3 g l^−1^) or with glucose (20 g l^−1^). After incubation, plates were stained for 30 min with amido black (3 g l^−1^ prepared in 3.5 M acetic acid) and washed with acetic acid to reveal clear halos around colonies if they possess degrading ability.

#### Mucin utilization

Using a method described previously [37], the log-phase cultures were inoculated in MRS broth (10 g l^−1^ proteose peptone, 10 g l^−1^ beef extract, 5 g l^−1^ yeast extract, 2 g l^−1^ ammonium citrate, 5 g l^−1^ sodium acetate, 0.1 g l^−1^ magnesium sulphate, 0.05 g l^−1^ manganese sulphate, 1 g l^−1^ Tween 80 and 2 g l^−1^ dipotassium phosphate) reconstituted with either glucose (20 g l^−1^) or mucin (3 g l^−1^). Cultures were aerobically incubated at 37 °C for 24 h, and OD was measured at 600 nm using a double-beam spectrophotometer (Labomed, USA). The results were expressed as growth (OD_600nm_) in mucin or glucose-reconstituted medium minus the residual growth in media with no fermentable carbohydrate source.

#### Bioaminogenic potential

The ability of the test strains to utilize amino acids and produce biogenic amines was assessed using a recommended method [38]. Log-phase cultures were sub-cultured twice in decarboxylating broth (HiMedia) supplemented with 0.1% (w/v) of amino acid, e.g. l-histidine monohydrochloride (Sigma-Aldrich), l-tyrosine disodium salt (Alfa Aesar, Germany), l-lysine monohydrate (Alfa Aesar, Germany) or l-ornithine monohydrochloride (Scharlau, Spain). Activated strains were then plated over decarboxylase agar substituted with 2% of their respective amino acid precursors and incubated aerobically at 37 °C for 72 h. Following incubation, formation of a purple zone indicated tyramine and a clear zone of histamine, putrescine or cadaverine.

### Screening the isolates based on antagonistic potential

#### Panel of indicator bacterial pathogens

The isolates were assessed for antimicrobial activity against selected Gram-negative bacterial pathogenic strains [*Escherichia coli* (ATCC25922) and *Pseudomonas aeruginosa* (ATCC15442)] and Gram-positive strains [*Bacillus cereus* (ATCC11778) and *S. aureus* (ATCC25923)], purchased from Microbiologics, USA. The strain *Salmonella enterica* serovar Typhi D1 was taken from the culture collection of the National Probiotic Lab (NPL) [39].

#### Hydrogen peroxide production

H_2_O_2_ production by bacterial isolates was determined using the colourimetric method [40]. Isolates were spotted over MRS agar plates supplemented with filter-sterilized HRP and tetramethylbenzidine. The plates were incubated at 37 °C for 40 h. Following incubation, colonies that produced H_2_O_2_ turned blue when exposed to air.

#### Antagonism assay

The well-established agar well diffusion method was used to estimate the strain’s antagonism towards select pathogen strains [38]. Cell-free supernatant (CFS) of tested strains was prepared by filter-sterilizing 48 h-old cultures. A freshly made soft nutrient agar (0.8%) plate was spread-plated with a culture of the select pathogenic bacterial isolate (10^8^ c.f.u. ml^−1^) and air-dried for 10 min; then, wells were punctured into using a sterile borer, and the well openings were sealed using 0.1% agar. CFS (100 µl) obtained from the cultures of the test isolates was added into the wells. The plates were refrigerated for 4 h before incubation and aerobically incubated at 37 °C. Sterile MRS broth acted as a negative control. The zone diameter of inhibition values in millimetres were subtracted from the well diameter and recorded. Antagonism was scored as ‘-’, ‘+’, ‘++’ and ‘+++’, indicating no zone (0 mm), weak (>0 but <3 mm), moderate (>3 but <6 mm) and strong zone (>6 mm), respectively.

#### Co-aggregation with select pathogenic bacteria

The ability to physically exclude pathogens was assessed via co-aggregation [41]. Log-phase cultures of pathogen strains and select isolates were separately harvested by centrifugation (4,500 ***g*** for 15 min). The bacterial pellets were washed twice with sterile PBS and resuspended in PBS to a density corresponding to OD_600nm_ of 0.25±0.05. Equal volumes (2 ml) of putative probiotics and pathogen cultures were mixed in a test tube and incubated statically at 37 °C. The OD_600nm_ of the upper layer was measured spectrophotometrically after 24 h, and co-aggregation (%) was calculated as follows:

Co-aggregation % = $\frac{\left(ODpro+ODpat\right)-ODmix}{\left(ODpro+ODpat\right)}\times 100$ (1)

OD_pro_, OD_pat_ and OD_mix_ represent the ODs of the test isolate, pathogen and mix suspension of probiotic and pathogen, respectively.

### Molecular identification of select isolates

Genomic DNA was extracted from pure cultures of the preserved isolates for molecular identification using the GeneJET Genomic DNA extraction kit (Thermo Scientific, Lithuania, EU). Adequate quality and quantity of the obtained DNA material were assessed spectrophotometrically using a NanoDrop (Thermo Scientific 2000C, Germany) and electrophoresis on a 1% agarose gel. The 16S rRNA universal primer pair (357F: 5′-CCTACGGGAGGCAGCAG-3′, 926R: 5′-CCGTCAATTCMTTTRAGT-3′) (Macrogen, Korea) was used for sequencing [42]. For PCR, each 10 µl reaction mixture contained 1 µl of DNA along with 0.2 µl (0.2 µM) of each of the forward and reverse, 0.2 µl of DNTPs (0.2 Mm), 1 µl buffer (1X), 1 µl MgCl_2_ and 6.1 µl of deionized water. The PCR was performed on Galaxy XP Thermal cycler with the conditions: initial denaturation at 95 °C for 10 min; 40 cycles of 95 °C for 1 min, 56 °C for 1 min and 72 °C for 1 min, with a final extension at 72 °C for 10 min. PCR products were analysed by electrophoresis on a 2% wt/vol agarose gel and sequenced on a BI3730XL 96-capillary DNA analyzer by Macrogen (Korea) using the same primer set above. The sequences were identified using blast algorithms and deposited in the NCBI’s GenBank for accession numbers (PP338758–PP338766).

### Characterizing the select strains for colonizing human gastrointestinal tract

#### Assessing the ability of select strains to persist in the human GIT

Tolerance to different human physiological gastrointestinal tract (GIT) stresses was tested using exponentially growing cultures of the strains. Such cultures were harvested by centrifugation (4500 *g* for 15 min), washed with sterile PBS, resuspended in the test media and processed as follows.

*Lysozyme resistance*: Resuspended cultures corresponding to a cell density of OD_600nm_ 0.1±0.05 were added to broth media carrying different concentrations of lysozyme (0 µg ml^−1^, 100 µg ml^−1^ and 200 µg ml^−1^). The cultures were then incubated, and their growth was compared with those growing without lysozyme, and given in terms of percentage survival rates [43].

*Acid resistance*: The strains were tested for their ability to tolerate stomach acidity using a known method [44]. Log-phase cultures of the test strains were resuspended in sterile PBS of varying acidities (pH 1.5, 3.0 and 7.0) to a cell density corresponding to OD 0.5±0.2 and incubated at 37 °C for 3 h. The stressed cultures were revived in 5 ml of fresh MRS broth, and differences in their growth were monitored spectrophotometrically after 5 h of incubation.

*Bile resistance*: The ability to tolerate bile, the primary intestinal stress agent in the test strains, was determined using a frequently used procedure [44]. Cultures of the test strains (OD ~0.1±0.05) prepared as above were exposed to bile mixed in freshly made MRS broth at various concentrations (0%, 0.15% and 0.3%). The growth of each culture was monitored after 5 h, and the results were interpreted based on differences in growth.

*Phenol resistance*: The resistance against phenol, a microbial toxic metabolite in the human GIT, was assessed using a slightly modified technique [43]. Test cultures (prepared as above) were suspended in fresh MRS broth supplemented with different phenol concentrations (0.2%, 0.4% and 0.6% w/v) to a starting cell density corresponding to 0.1±0.05. The OD was measured hourly up to 10 h using a SpectraMax Plus 384 microplate reader.

Sterile growth media were used as a negative control, whereas unstressed cultures in growth media acted as a positive control.

#### *In vitro* assessment of adhesion ability

*Autoaggregation*: To what degree the bacterial cell’s surface is conducive for good adhesion was assessed *in vitro* using a widely used technique [41]. The strains were cultured in MRS broth medium at 37 °C till their log phase (~4 to 6 h) and harvested by centrifugation (5,000 ***g*** for 15 min), washed thrice and suspended in 5 ml sterile PBS (0.5±0.05). The bacterial suspensions were statically incubated at 37 °C, self-clumping was monitored hourly for 5 h and the final reading was taken at 24 h. The auto-aggregation percentages were calculated as follows, and the results were interpreted as strong (>50%), moderate (20%–50%) and weak (<20%).

% Auto aggregation = $\frac{OD0-ODt}{O.D0}\times 100$ (2.6)

Where OD_t_ and OD_o_ are OD_600nm_ measurements taken at time t and 0 h.

*Bacterial adhesion to hydrocarbon*: the tendency of the strains to adhere was estimated *in vitro* based on their affinity for xylene [41]. Log-phase culture suspensions (0.5±0.05), as described above, were prepared and mixed (1 : 1) with pure xylene. Following the separation of the aqueous and solvent phases, the OD_600 nm_ of the aqueous layer (OD_t_) was compared with that of the culture suspension (OD_o_=0.5±0.05) using the following equation:

% Hydrophobicity = $\frac{OD0-ODt}{OD0}\times 100$ (2.7)

Hydrophobicity percentages were recorded as strong (>50%), moderate (20%–50%) and weak (<20%) hydrophobic.

*Mucin adhesion*: The ability of bacteria to adhere to mucin was assessed using standard techniques [45]. The mucin-coated plates were prepared with 10 mg ml^−1^ of gastric mucin (from porcine stomach, type II, Merck) and saturated with BSA solution (20 mg ml^−1^). The bacterial suspensions (0.1±0.05) prepared as above in PBS were transferred to mucin-coated wells and incubated at 37 °C for 2 h. The wells were emptied and washed twice with sterile PBS to remove unattached cells. Adhered bacterial cells were then detached by treating them for 2 min with a 0.25% trypsin/EDTA solution (Corning, NY, USA), resuspended in PBS, and OD at 600 nm was measured using a Microplate SpectraMax Plus 384 plate reader.

*Biofilm formation*: The biofilm potential of the individual strains was assessed using the classical crystal violet assay employing 96-well polystyrene plates, whose wells were either mucin-coated or uncoated [46]. For coating, a filter-sterilized mucin solution (1 mg ml^−1^ of porcine stomach type II from Merck dissolved in sterile DW) was added to each well and dried overnight under refrigerated conditions. Log-phase cultures of the test strains corresponding to a density of 0.01±0.05 were added to the wells in triplicates and allowed to grow till 72 h at 37 °C. The bacterial cultures were carefully pipetted, and the wells were gently washed with sterile PBS. The wells were then flooded with 200 µl of crystal violet (0.1% w/v) and kept at ambient temperature for 30 min. The stain retained within the biofilm material was dissolved in pure dimethyl sulphoxide, and the absorbance was measured at 570 nm using a Microplate SpectraMax Plus 384 plate reader. A sterile medium without the added bacterial inoculum acted as the negative control.

### Identifying functional attributes of select strains

#### Antioxidant activity

The antioxidative capacity of the selected strains was determined using two assays, namely, the hydroxyl radical scavenging (HRS) and the superoxide anion scavenging (SAS) [47].

*HRS assay*: 1,10-Phenanthroline and FeSO_4_ (6 mmol l^−1^) were mixed in equal amounts and immediately diluted by adding 1 ml of sterile PBS. Freshly prepared bacterial cultures were added, followed by a 500 µl solution of H_2_O_2_ (0.1% v/v). The same solution without bacterial suspension was considered the negative control, while the solution containing only 1,10-phenanthroline and FeSO_4_ was served as blank. Then, sterile water was added to make the volume up to 4 ml. Two hundred microlitres of this solution were dispensed into the wells of a 96-well microtitre plate, and the OD_536nm_ was measured using the SpectraMax Plus 384 microplate reader, which was used for calculating:

Hydroxyl radical scavenging (%) = $\frac{As-A1}{A0-A1}\times 100$ (2.8)

Where As is the sample absorbance, A1 is the control absorbance and A0 is the blank absorbance.

*SAS assay (pyrogallol autoxidation)*: Log-phase cultures suspended in PBS (50 µl) were diluted with 910 µl of sterile water and immediately mixed with 2 ml Tris–HCl (50 mmol l^−1^, pH 8.2) buffer. Pyrogallol solution (40 µl, 10 mmol l^−1^) was then added. Autoxidation was then measured as the maximum inhibition point of reaction by taking absorbance every 1 min for 10 min using a SpectraMax Plus 384 microplate reader. Deionized water in place of cell suspension was used as a control, and the results were calculated using the formula:

Superoxide anion scavenging activity (%) = $\frac{\Delta Ao-\Delta A}{\Delta Ao}\times 100$ (2.9)

The symbol ΔAo denotes the pyrogallol rate of autoxidation before, and ΔA represents the pyrogallol rate after adding the sample and water.

#### Bile salt hydrolase activity

The ability of bacterial isolates to hydrolyze the bile salt was determined by a method described elsewhere [48]. Specialized media plates were prepared by supplementing MRS (Merck, Germany) agar with 0.5% (w/v) of four different bile salts: tauroglycocholate (HiMedia, India), taurocholate (HiMedia, India), taurodeoxycholate (Merck, Sigma-Aldrich), deoxycholate (HiMedia, India) and 0.37 g l^−1^ of CaCl_2_. Freshly grown bacterial cultures were spot-plated (2 µl) on pre-labelled agar plates and incubated at 37 °C for 48 h. The precipitation zones arising from bile salt hydrolysis around the colonies were recorded as evidence of bile salt hydrolase (BSH) function. The diameter of the zone was measured (millimetres) using a measuring scale.

#### Cholesterol assimilation

Test strains were examined to determine whether they could assimilate cholesterol using an established methodology [41]. Freshly made MRS supplemented with cholesterol PEG (Sigma-Aldrich, India) at 100 µg ml^−1^ broth was inoculated with overnight cultures and allowed to grow for 48 h at 37 °C. The assimilated cholesterol was determined based on the amount of cholesterol that remained in the supernatants. The CFLs (500 µl) were mixed with 500 µl of 33% (w/v) KOH solution and 1 ml absolute ethanol and vortex-mixed before being incubated at 37 °C for 15 min. The mixture was diluted using 1 ml of deionized water and mixed with 1.5 ml of hexane. The unspent cholesterol was captured in a hexane layer, then separated carefully and evaporated. Following complete evaporation, o-phthalaldehyde (50 mg dl^−1^) reagent prepared in acetic acid was added, followed by H_2_SO_4_ (250 µl) and incubated at 37 °C for 20 min. Absorbance was measured at OD_570_ nm, and assimilated cholesterol was quantified using a standard curve prepared with different cholesterol concentrations (0, 20, 40, 60, 80 and 100 µg ml^−1^) and plotted against the OD_570_ nm values as reported elsewhere.

#### Carbohydrate utilization ability

HiCarbo kit (HiMedia, India) was used per the manufacturer’s instructions to determine the test strain’s ability to hydrolyze di- and monosaccharides enzymatically. Log-phase cultures (50 µl) were inoculated aseptically in the kit’s cupules and incubated at 37 °C for 24 h, and the colour changes were visually noted.

#### Exopolysaccharide production

The ability to produce exopolysaccharide (EPS) under optimal cultivation conditions was estimated using an established methodology [49]. Activated cultures (4% inoculum v/v) were inoculated in 10 ml MRS broth and incubated for 48–72 h at 37 °C, following which the cultures and enzymes were deactivated by boiling for 15 min, then cooled to room temperature (25 °C), and concentrated trichloroacetic acid (85% w/v) was added (17% v/v) to them at room temperature, and the mixture was thoroughly homogenized. The bacterial cells were separated by centrifugation at 13,000 ***g*** for 30 min at 4 °C. The supernatant was mixed with ethanol (1 : 1), and a second ethanol precipitation was applied. The precipitated EPS–protein complex was pelleted down centrifugally (13,000 ***g*** for 14 min at 4 °C) and kept on the lab bench for 30 min. Then, this was dissolved in sterile DW, and EPS was estimated using the phenol-sulphuric acid method. For this, a homogeneous suspension was vortexed with 50 µl of phenol. Sulphuric acid (50 µl) was added slowly to complete the volume up to 2 ml. Samples were left for 10 min and shaken thoroughly. About 200 µl of the sample was loaded in the wells of a 96-well microtitre plate, and absorbance was measured spectrophotometrically at 480 nm using a SpectraMax Plus 384 microplate reader. Quantification was done using a 7-point calibration curve of a glucose stock solution (50 mg ml^−1^).

#### Safety profiling of select strains based on antibiotic susceptibilities

The broth micro-dilution approach was used to determine susceptibilities towards nine frontline antimicrobials specified in the guidelines of the Clinical and Laboratory Standards Institute (CLSI 2018). The antimicrobials used were ampicillin (TSI) (0.125–8 µg ml^−1^), vancomycin (Bio Basic) (0.25–16 µg ml^−1^), gentamicin (Bio Basic) (2–128 µg ml^−1^), kanamycin (Bio Basic) (2–128 µg ml^−1^), streptomycin (Bio Basic) (2–128 µg ml^−1^), erythromycin (Bio Basic) (0.125–8 µg ml^−1^), clindamycin (TSI) (0.125–8 µg ml^−1^), tetracycline (Bio Basic) (0.5–32 µg ml^−1^) and chloramphenicol (TSI) (0.5–32 µg ml^−1^). The strains were grown in MRS broth until their log phase at 37 °C. Fixed concentrations of antibiotics were added (50 µl) to each well of a 96-well microplate (Sarstedt, Nümbrecht, Germany), followed by adding 50 µl of log-phase cultures of the test strains in MRS media. The final volume was raised to 200 µl by adding sterile MRS broth. Bacterial cultures grown in MRS broth without antibiotics were taken as a positive control, while the uninoculated MRS broth was the negative control. The plates were incubated aerobically at 37 °C for 24–48 h, and the minimum concentration of antibiotics that inhibited bacterial growth was determined. Finally, the strains were classified as resistant or susceptible to a specific antibiotic depending on the European Food Safety Authority (EFSA) microbiological cut-off concentration for that particular antibiotic [50].

### Testing survival of the best candidate strain under simulated human GIT conditions using the COST-INFOGEST *in vitro* digestion model

The survival of the selected strain was examined under simulated human physiological upper GIT conditions using an *in vitro* INFOGEST digestion model [51]. The test strain was subjected to sequential oral, gastric and intestinal digestion, while parameters such as enzymes, pH, electrolytes, bile, dilution and digestion time were set to reflect normal human physiological conditions.

The simulated digestive fluids, including salivary fluid (SSF), simulated gastric fluid (SGF) and simulated intestinal fluid (SIF), were prepared in electrolyte stock solutions of enzymes, CaCl_2_ and water. On the day of the experiment, the organic and inorganic solutions were mixed and supplemented with digestive constituents. Saliva was supplemented with filter-sterilized uric acid (10 mg/5 ml) and mucin (10 mg/5 ml). SGF contains pepsin (12.3 mg/10 ml), BSA (20 mg/10 ml) and mucin (200 mg/2 ml), and SIF was supplemented with BSA (40 mg/2.5 ml), lipase (20 mg/2.5 ml), pancreatin (30 mg/5 ml) and bile (60 mg/5 ml). The digestive juices were made and maintained at 37 °C, the average human body temperature.

Log-phase bacterial suspensions having cell density (3×10^9^ c.f.u. ml^−1^) were stressed (1 : 1) with SSF (pH 7) for 2 min. Next, the bolus suspension was mixed (1 : 1) with SGF (pH 3) and pepsin for 120 min. As a final step, the gastric chyme was mixed (1 : 1) with SIF containing bile (10 mM l^−1^) and pancreatin (100 U trypsin activity per millilitre). The SGF and SIF incubations were done anaerobically at 37 °C. Unstressed (buffer solution without organic parts) bacterial growth was used as a positive control. Sample aliquots were taken before and after every digestion treatment, plated on MRS and enumerated (c.f.u. per millilitre). Survival rates were calculated and recorded for further analysis.

### Statistical analysis

Experiments were replicated thrice, and data were expressed as means±sd. One-way ANOVA and Tukey’s multiple comparisons were used to test for significant differences (*P*<0.05) between means. The principal component analysis (PCA) was applied to probiotic and functional datasets using XLSTAT (v. 2020.1.1, Addinsoft, USA). We used K-means algorithms that use kernels to estimate the distance between dataset values.

## Results

### Isolations from fermented beverages and their primary bacteriological characterization

**Fig. 1.**
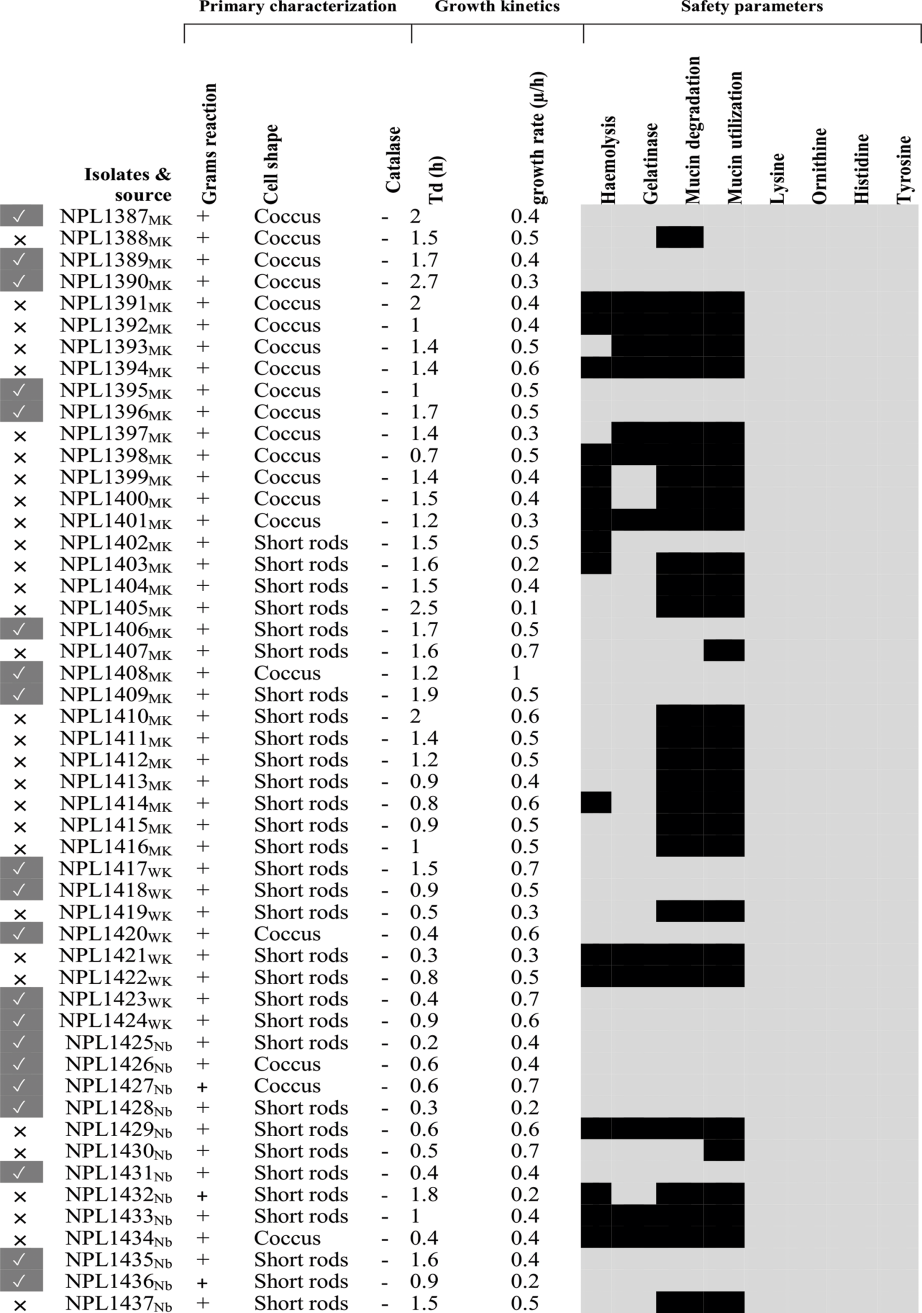
Primary biochemical characteristics, growth kinetics and safety parameters of isolates obtained from the lab-prepared fermented beverages. Black colour indicates positive; white colour means negative.

Bacterial diversity was greater in milk-fermented products than in water-based ones. A total of 30 colonies from milk kefir, 8 from water kefir and 13 from nabeez were selected and preserved. All isolates were Gram-positive; the cells were either coccus (39%) or short rods (61%). All the isolates from the three fermented products examined were catalase-negative. When grown aerobically in MRS broth, milk kefir cultures showed a more extended lag phase (Fig. 1). On average, strains from water kefir grew faster than the milk ones. All isolates' growth rate (µ/h) ranged from 0.13 to 0.99, and the T_d_ was between 0.24 and 2.69 per hour.

### Safety status

The safety of the strains was assessed *in vitro* using a classical culture-dependent approach and presented in Fig. 1. Most of the milk-based kefir isolates (24 in total) tested were unsafe for human use, with at least one undesirable enzymatic activity. Alpha haemolysis was notable in ten isolates, half of which were also positive for gelatinase production. The isolates with substantial gelatinase activity but no haemolysis were NPL1393_MK_ and NPL1397_MK_. The ability to break down mucin was found in 20 strains, 18 of which could also use this for their growth. When used as the only carbon source in growth media, all mucin-degrader strains could digest mucin, with strain NPL1407_MK_ being the exception. Interestingly, NPL1388, a mucin-degrading strain from milk kefir, could not consume free mucin. Strains from water-based fermented products also raised safety concerns, but these were minor. Approximately 62% and 54% of water kefir and nabeez strains were deemed safe, respectively. All alpha haemolytic strains of water-based beverages were also gelatinase positive except for NPL1432_Nb_. Three strains of water kefir and four of nabeez were mucin degraders and could also metabolize it. NPL1430_Nb_ also demonstrated mucin utilization ability. Biogenic amine production was absent in all isolates of three fermented products (Fig. 1).

Of the 51 bacterial isolates from the fermented products, more than half (~30) demonstrated enzymatic activity linked with safety concerns and were excluded from further testing. The remaining 20 isolates, 8 from milk kefir, 5 from water kefir and 7 from nabeez, were then checked for their antimicrobial abilities.

### Antagonistic potential

The antimicrobial activity was checked via physical- (co-aggregation) and chemical-exclusion (well diffusion and H_2_O_2_ production) methods, and the results were subjected to hierarchical cluster analysis (HCA) (Table 1). The statistical method used divided the test strains into two major clusters based on the similar characteristics. Five milk kefir, one nabeez and four water kefir strains could kill or exclude the test pathogens and clustered independently. Overall, the milk kefir strains, except for NPL1402 and NPL1409, had good antagonism and outperformed those from the water-based beverage. The strain NPL1402_MK_ showed considerable co-aggregation with *S. aureus* and *P. aeruginosa*, but their weak aggregation with other pathogens and dismal performance in well diffusion led them to group the other poorly performing candidates. The strain NPL1409_MK_ was moderately effective in both cases. The antimicrobial trend was reduced in strains produced from water kefir, with only one strain (NPL1420) showing significant antagonistic potential. Among the strains recovered from nabeez, NPL1426 stood out for its co-aggregation and antibacterial capabilities.

**Table 1.** Antimicrobial activity of fermented beverage isolates and their HCA

‘−’: >10; ‘+’: 11–40; ‘++’: 40–70; ‘+++’: >70.

*Species not identified.

B.c, *B. cereus*; E.c, *E. coli*; MK, milk kefir; Nb, nabeez; P.a, *P. aeruginosa*; *S. a*, *S. aureus*; *S. t*, *S. typhi*; WK, water kefir.

### Molecular identification of the outstanding isolates

**Fig. 2.**
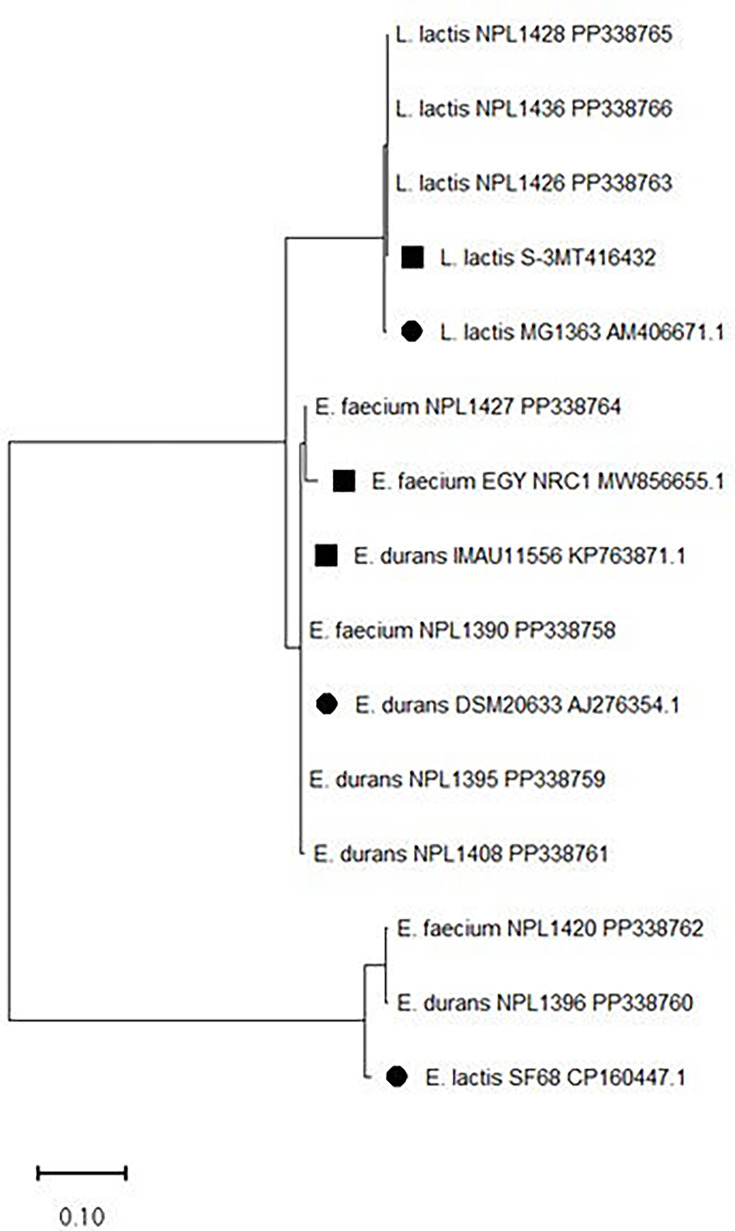
Genetic relatedness based on 16S rRNA of strains derived from fermented beverages and reference strains was inferred using the maximum-likelihood method and the Jukes–Cantor model. Reference strains used are (●) probiotic type strains and (■) probiotic strains isolated from dairy origin. Evolutionary analysis was conducted in mega11.

Select strains were molecularly identified as belonging to *Enterococcus faecium*, *Enterococcus durans* and *L. lactis* species. Based on sequence similarities of 16S rDNA, *E. faecium* strain NPL1420 from water kefir and *E. durans* strain NPL1396 from milk kefir were close, whereas the *L. lactis* strains clustered separately (Fig. 2). All the identified strains had notable antimicrobial potentials and were further evaluated for functional and probiotic properties.

### Assessing probiotic aptitude

#### Tolerance to human gut physiological conditions

**Fig. 3.**
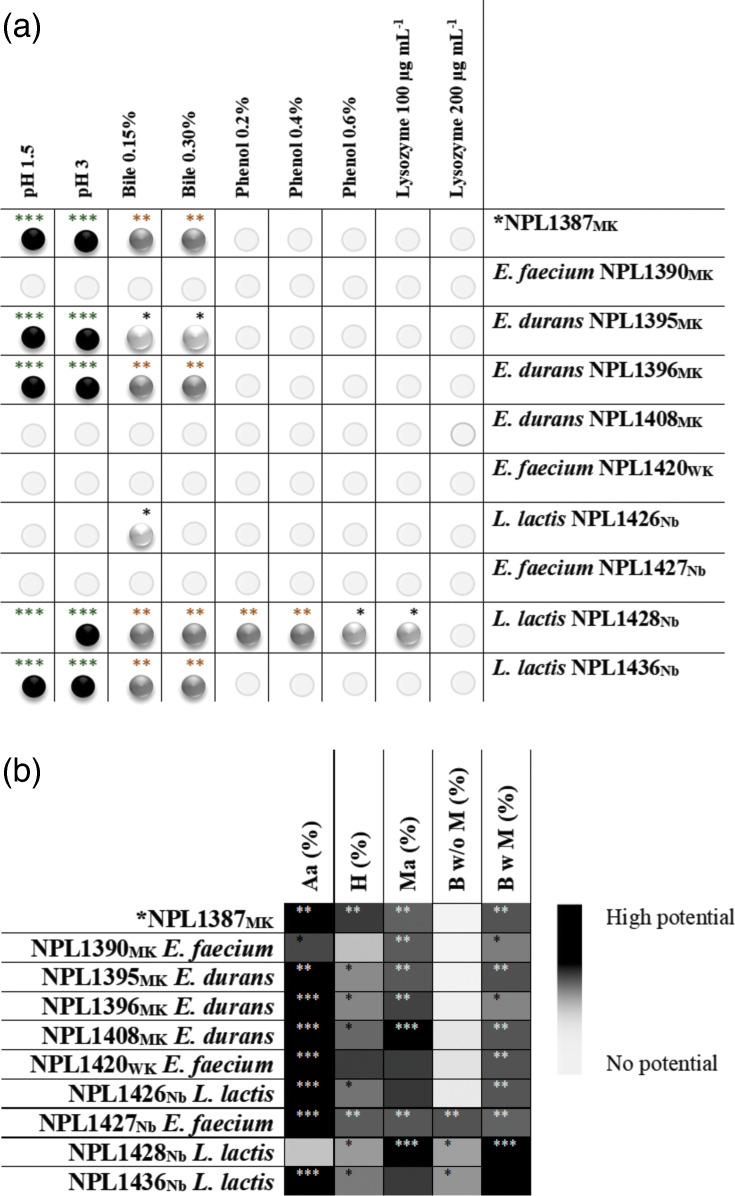
Probiotic potential testing of strains of fermented beverage origin. *Species of the isolate remains unidentified. (**a**) GIT stress tolerance of strains (*** statistically significant; significant tolerance, moderate tolerance, poor tolerance and no tolerance). (**b**) Adhesion capacity of the strains. (Aa: autoaggregation; H: cell surface hydrophobicity; Ma: mucin adhesion; B w/o M: biofilm in the absence of mucin layer; B w M: biofilm in the presence of mucin layer).

Fig. 3(a) depicts the ability of the strains to survive the human gut’s physiological stressors and their adhesion potential. Only three milk kefir strains and two nabeez strains could withstand high acidity. However, all milk kefir and nabeez strains were relatively tolerant of increased bile concentrations. Resistance towards phenol and lysozyme was generally absent except in strain NPL1428_Nb_, which was moderately tolerant of phenol and lysozyme concentrations (100 µg ml^−1^). The water kefir strain NPL1420 did not grow properly under all tested physiological parameters.

#### *In vitro* assessment of adhesion potential

The strains showed good adhesion and biofilm potential in the *in vitro* tests (Fig. 3b). Except for the NPL1428_Nb_ strain, the rest demonstrated significant autoaggregation. In general, strains with good cell surface hydrophobicity and autoaggregation adhered better to mucin. Interestingly, biofilm development was strong in *L. lactis* strain NPL1428, which originated from nabeez, and much more significant on mucin-coated surfaces despite the poor autoaggregation. Overall, the nabeez-derived strains outperformed the rest regarding adherence to inanimate surfaces. However, only one nabeez-origin *E. faecium* strain, NPL1427, produced a good biofilm (~60%) on uncoated or mucin-coated polystyrene surfaces.

### Screening of functional attributes

The gamut of functional attributes displayed by the strains recovered from the fermented beverages is depicted in Table 2. All strains showed good antioxidant activity, especially the ones from nabeez. Except for the water kefir strain (NPL1420) and nabeez strain (NPL1426), which had a limited ability to digest cholesterol, the rest performed well regarding free cholesterol absorption. The ability of *E. duran*s and *L. lactis* strains to hydrolyze bile salts was notable. EPS production was significant in all strains of *E. faecium*, *E. durans* and *L. lactis*.

**Table 2.** Functional characterization of strains isolated from fermented beverages

Strains and source	Antioxidant potential		Cholesterol assimilation %	EPS production (mg l^−1^)	BSH activity
	HRS %	SAS %			
NPL1387_MK_*	55±0.02^b^	39±0.11^a^	85±0.03^c^	437±0.03^a^	−
***E. faecium*** **NPL1390_MK_**	60±0.05^b^	40±0.03^a^	90±0.04^d^	538±0.02^c^	−
***E. durans*** **NPL1395_MK_**	32±0.10^a^	61±0.04^b^	90±0.04^d^	554±0.05^d^	+
***E. durans*** **NPL1396_MK_**	98±0.04^e^	71±0.06^c^	93±0.06^d^	453±0.07^a^	−
***E. durans*** **NPL1408_MK_**	76±0.00^d^	76±0.06^c^	83±0.09^c^	560±0.02^d^	+
***E. faecium*** **NPL1420_WK_**	68± 0.11^c^	87±0.07^d^	43±0.12^a^	429±0.11^a^	−
***L. lactis*** **NPL1426_Nb_**	55±0.09^b^	83±0.04^d^	56±0.03^b^	560±0.13^d^	−
***E. faecium*** **NPL1427_Nb_**	69±0.04^c^	56±0.01^b^	82±0.01^c^	504±0.04^b^	−
***L. lactis*** **NPL1428_Nb_**	94±0.02^e^	78±0.11^c^	78±0.02^c^	595±0.09^e^	+
***L. lactis*** **NPL1436_Nb_**	95±0.01^e^	63±0.10^b^	84±0.07^c^	599±0.09^e^	+

*Species not identified.

Results are expressed as the mean±sd (*n*=3). The letters correspond to the Tukey test results (*P*<0.05), and different letters in each column indicate a significant difference in the Tukey test.

MK, milk kefir; Nb, nabeez; WK, water kefir.

The profile of the strains with active carbohydrate-acting enzymes involved in fermenting diverse substrates is presented in Table 3. The ability to ferment sorbitol, dulcitol and *α*-methyl-d-mannoside is unique to milk kefir strains, whereas nabeez strains were prominent in sorbose fermentation. *L. lactis* NPL1436_Nb_ was the only strain that fermented the monosaccharidic sugar mannose.

**Table 3.** Carbohydrate fermentation profile of strains isolated from fermented beverage

Sugars	^a^*NPL1387_MK_	^a^*E. faecium* NPL1390_MK_	^a^*E. durans* NPL1395_MK_	^a^*E. durans* NPL1396_MK_	^b^*E. durans* NPL1408_MK_	^c^*E. faecium* NPL1420_WK_	^d^*L. lactis* NPL1426_Nb_	^d^*E. faecium* NPL1427_Nb_	^d^*L. lactis* NPL1428_Nb_	^e^*L. lactis* NPL1436_Nb_
Lactose	+	+	+	+	+	+	+	+	+	+
Xylose	+	+	+	+	+	+	+	+	+	+
Maltose	−	−	−	−	−	−	−	−	−	−
Fructose	+	+	+	+	+	+	+	+	+	+
Dextrose	+	+	+	+	+	+	+	+	+	+
Galactose	+	+	+	+	+	+	+	+	+	+
Raffinose	−	−	−	−	−	−	+	+	+	+
Trehalose	−	−	−	−	−	+	+	+	+	+
Melibiose	+	+	+	+	+	+	+	+	+	+
Sucrose	−	−	−	−	−	−	−	−	−	−
l-Arabinose	+	+	+	+	+	+	+	+	+	+
Mannose	−	−	−	−	−	−	−	−	−	+
Inulin	+	+	+	+	+	+	+	+	+	+
Na gluconate	+	+	+	+	+	+	+	+	+	+
Glycerol	+	+	+	+	+	+	+	+	+	+
Salicin	+	+	+	+	+	+	+	+	+	+
Dulcitol	+	+	+	+	−	−	−	−	−	−
Inositol	+	+	+	+	+	+	+	+	+	+
Sorbitol	+	+	+	+	−	−	−	−	−	−
Mannitol	+	+	+	+	+	+	+	+	+	+
Adonitol	+	+	+	+	+	+	+	+	+	+
Arabitol	+	+	+	+	+	+	+	+	+	+
Erythritol	+	+	+	+	+	+	+	+	+	+
*α*-Methyl-d-glucoside	−	−	−	−	−	−	−	−	−	−
Rhamnose	−	−	−	−	−	−	−	−	−	−
Cellobiose	+	+	+	+	+	+	+	+	+	+
Melezitose	+	+	+	+	+	+	+	+	+	+
*α*-Methyl-d-mannoside	+	+	+	+	+	−	−	−	−	−
Xylitol	+	+	+	+	+	+	+	+	+	+
ONPG	−	−	−	−	−	−	−	−	−	−
Aesculin	+	+	+	+	+	+	+	+	+	+
d-Arabinose	+	+	+	+	+	+	+	+	+	+
Citrate	−	−	−	−	−	−	−	−	−	−
Malonate	−	−	−	−	−	−	−	−	−	−
Sorbose	−	−	−	−	−	+	+	+	+	+

+, positive to ferment sugar; −, no sugar fermentation.

MK, milk kefir; Nb, nabeez; WK, water kefir.

Antibiotic resistance is a significant issue of concern and an unwanted attribute in probiotic candidate strains. The tested strain’s resistance and susceptibility patterns to select antibiotics are presented in Table 4. All of them were susceptible to aminoglycosides, i.e. gentamicin and streptomycin, except for *E. durans* NPL1396_MK_, which could resist very high concentrations (1024 µg ml^−1^). Unlike the strains of Nabeez, where only one strain, NPL 1436, harboured resistance to ampicillin, it was ubiquitous in those derived from milk kefir. Chloramphenicol resistance was present in all strains obtained from water-based kefir beverages but in only one milk kefir origin strain (NPL1395).

**Table 4.** Antibiotic resistance profile of strains isolated from fermented beverages using MIC

Strains and source	Ampicillin (µg ml^−1^)	Gentamycin (µg ml^−1^)	Streptomycin (µg ml^−1^)	Erythromycin (µg ml^−1^)	Clindamycin (µg ml^−1^)	Tetracycline (µg ml^−1^)	Chloramphenicol (µg ml^−1^)
*Enterococcus*	2	32	128	4	4	4	16
*L. lactis*	2	32	32	1	1	4	8
NPL1387_MK_*	16^R^	4^S^	64^S^	0.5^S^	4^S^	4^S^	2^S^
*E. faecium* NPL1390_MK_	16^R^	4^S^	16^S^	2^S^	2^S^	8^R^	16^S^
*E. durans* NPL1395_MK_	16^R^	16^S^	16^S^	0.5^S^	32^R^	0.5^S^	128^R^
*E. durans* NPL1396_MK_	0.25^S^	32^S^	1024^R^	32^R^	2^S^	4^S^	2^S^
*E. durans* NPL1408_MK_	8^R^	16^S^	16^S^	0.125^S^	8^R^	0.5^S^	0.5^S^
*E. faecium* NPL1420_WK_	0.125^S^	2^S^	16^S^	0.125^S^	1^S^	0.5^S^	32^R^
*L. lactis* NPL1426_Nb_	1^R^	4^S^	2^S^	8^R^	8^R^	0.5^S^	32^R^
*E. faecium* NPL1427_Nb_	0.125^S^	64^S^	128^S^	32^R^	8^R^	32^R^	32^R^
*L. lactis* NPL1428_Nb_	1^S^	32^S^	128^S^	0.5^S^	0.5^S^	1^S^	32^R^
*L. lactis* NPL1436_Nb_	8^R^	8^S^	16^S^	8^R^	0.5^S^	8^R^	32^R^

*Species not identified.

S: susceptible; R: resistant according to EFSA breakpoints.

MK, milk kefir; Nb, nabeez; WK, water kefir.

Interestingly, the water kefir strain (NPL1420) was susceptible to all but chloramphenicol. Antibiotic resistance was more frequent in nabeez strains than those derived from the other two test beverages. Resistance in *L. lactis* strains was variable; NPL1436 was resistant to five of the tested antibiotics, whereas another strain, NPL1428, was resistant to chloramphenicol only.

### Strain selection using PCA

**Fig. 4.**
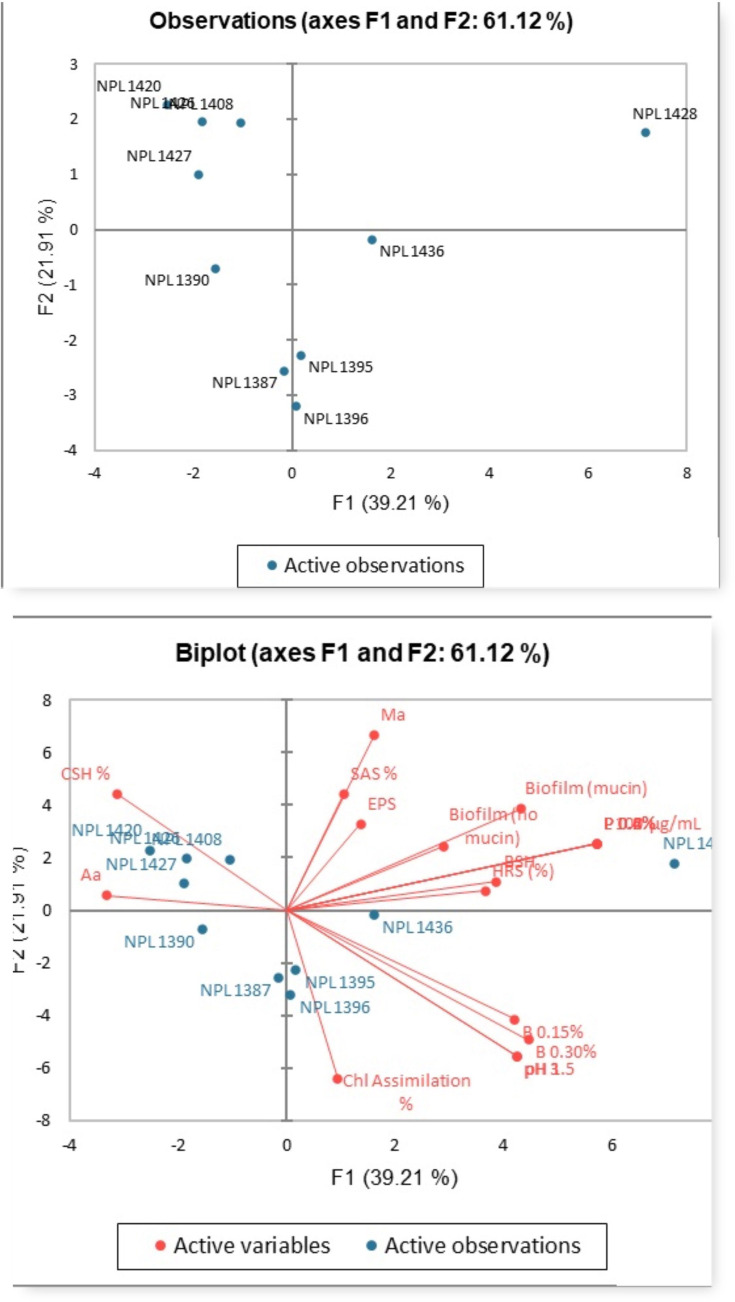
Graphical representation of the correlation biplots using PCA. (**a**) Active observations (tested probiotic strains). (**b**) Projection of active variables (probiotic characterization assays) in two-dimensional planes and their relation to active observations.

The probiotic functionalism of the test strains was also statistically examined using PCA to identify the best probiotic contender. The biplot graphs on PCA are presented in Fig. 4. The maximum variability covered by the first two principal components was 61.12%, with F1 and F2 accounting for 39.21% (7.058 eigenvalues) and 21.19% (3.944 eigenvalues) of the variation. Only cell surface hydrophobicity and autoaggregation were in the negative region of PC1. Strain-specific differences in probiotic attributes were seen among the tested candidates. PCA analysis showed that only the strain NPL1428_Nb_ was more linked with improved adhesion, biofilm formation, GIT tolerance to phenol and lysozyme, antioxidant potential and EPS generation.

### Survival under simulated human GIT conditions

**Fig. 5.**
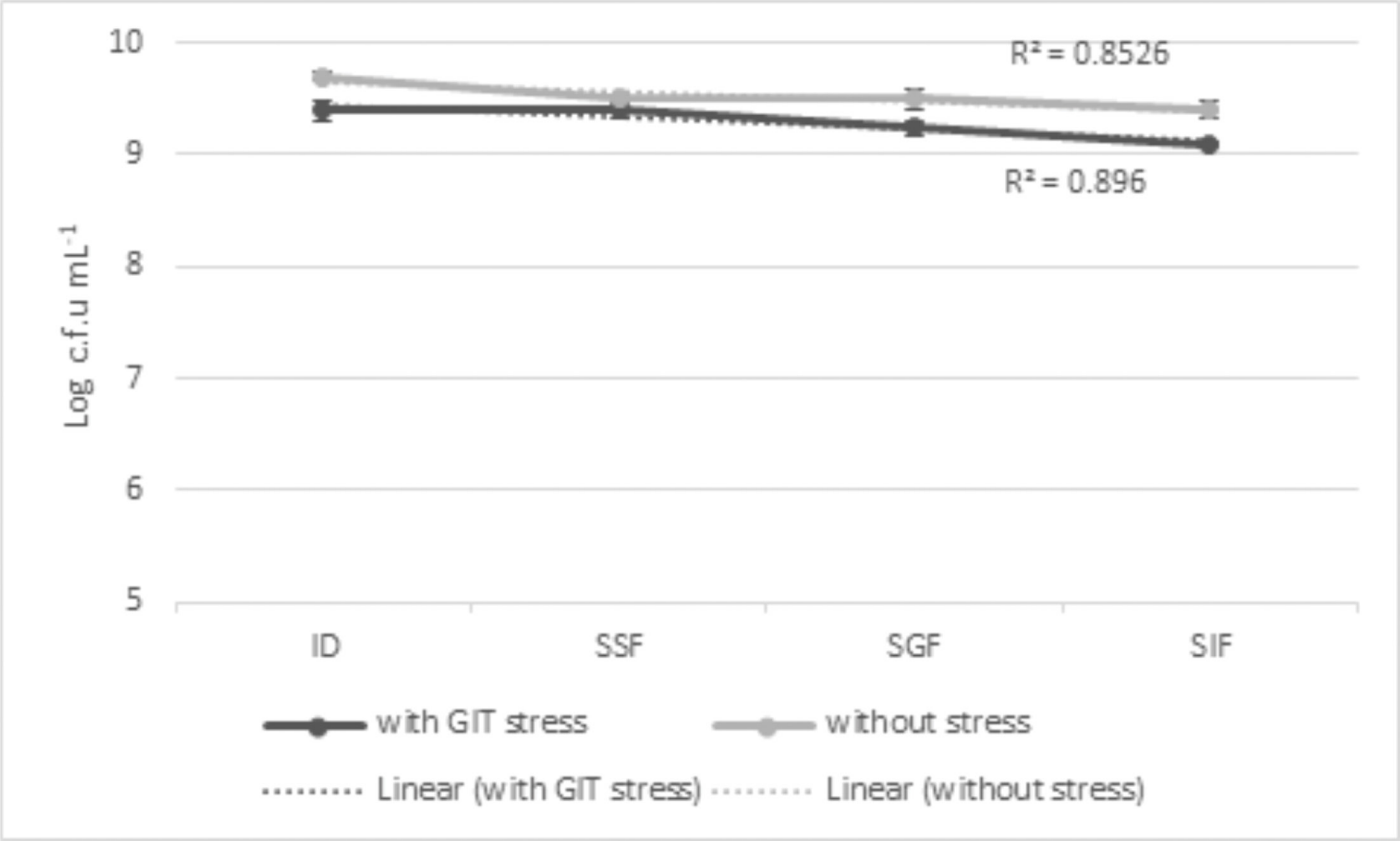
Survivability of the *L. lactis* strain NPL1428_Nb_ under simulated GIT stresses using the static COST-INFOGEST model.

The *L. lactis* NPL1428 strain demonstrating outstanding probiotic and functional aptitudes was chosen to check its survivability using the INFOGEST digestion model presented in Fig. 5. The studied strain withstands all stressors with only a slight drop in viability (~0.6 log c.f.u. reduction). Salivary fluids had little effect on the strain’s viability, and only a modest reduction of 0.2 log was seen after simulated stomach acid exposure. Bile was discovered to be most detrimental to this strain, causing the viability to decrease by 0.6 log c.f.u.

## Discussion

Traditional fermented foods and beverages (TFFB) with a long history of use in human cuisine are popular for their positive health effects [52]. Many studies have linked the microbiota of fermented food or beverages as responsible for these health effects [52]. This study focused on the regional TFFB as a source of potentially probiotic bacterial strains.

Kefir, a widely consumed fermented product, is famous for its health effects due to its unique nutritional, chemical and microbial composition [53,54]. Kefir has gained considerable popularity in recent years with many domestic consumers sharing and propagating its grains. Kefir’s long history of use and its stable microbial community make it well-suited for making innovative functional beverages utilizing a variety of dairy, preferably though non-dairy substrates are also an option [2,4, 13, 55]. The global market for kefir is growing due to its therapeutic and nutritional benefits [2,3, 5], encouraging a shift in its production from artisanal cottage industry to large-scale industrialized production involving starter cultures [13]. Isolating and standardizing microbes for kefir production is an important research goal [2,4]. Nabeez, a popular Middle Eastern fermented beverage made from dates, is distinctive in quality due to many fermentable sugars in it that promote microbial diversity. Date palm sugars are a favourable substrate for LAB spp., including the fructophilic ones [56]. The limited research done on date palm so far identifies its sugar content as primarily responsible for maintaining its microbiota. Its medicinal value stems from its presumed growth-promoting effect on beneficial bacteria [57].

The preferred approach for assessing bacterial diversity combines culture-based and molecular approaches [11]. Agar-based culturing continues to be the preferred choice when it comes to isolating novel probiotic strains from complex environments [58,59]. The spread-plate technique used here allows for discrete and easily accessible microbial colonies, which aids in the pure culturing of isolates [60]. Spreading samples on agar media plates and cultivating them aerobically are widely employed in studies aimed at assessing bacterial prevalence and diversity in any niche [28,61]. Milk-fermented beverages generally have more diverse bacterial content because of the abundance and accessibility of nutrients compared to the fewer nutritional substrates in water-based ones [7]. Predictably, the low number of bacteria retrieved from water kefir could be owing to their concentration within the grains rather than being dispersed and distributed in water [6]. The literature on the microbial makeup of date fruit is limited, but enriching functional beverages with dates does boost levels of probiotics [62,64]. Date palm syrup is purportedly an economical option for elevating probiotic numbers in fruit juices [65]. Nevertheless, the microbial composition of the final product is influenced by the substrate, grain source and incubation conditions [4,12, 53].

The safety aspects of these strains derived from fermented beverages were assessed to ensure their acceptability for human consumption. Understanding the microbial community of natural food products is imperative for standardizing preparation methods and improving product quality and safety [3]. The guidelines put forth by the Food and Agriculture Organization/World Health Organization (FAO/WHO) clearly stipulate a set of essential safety prerequisites that any test probiotic micro-organism must fulfil [66]. Virulence factors, like haemolysin and gelatinase, enhance a bacterium’s ability to cause disease and figure prominently in the probiotic safety assessment [67]. An inability to haemolyze blood by strains of *Enterococcus* species demarcates harmful from benign enterococci [66,68, 69], and this must be incontrovertibly established using *in vitro* tests [70]. An absence of haemolytic activity, as demonstrable in some of the strains studied here, suggests their suitability for human use because such strains are decidedly non-cytotoxic and incapable of causing bloody diarrhoea in humans [20]. Another bacterial enzyme that evokes safety concern is gelatinase, which can degrade extracellular matrix materials including various types of proteins. It helps spread infection by enabling the pathogen to invade the host tissues [71]. An actively functioning gelatinase can cause endocarditis and disturb the host’s immune system [71]. *Enterococcus* species sourced from fermented foods generally lack gelatinase [72], as is the case here. Some bacterial strains impart their pathogenicity by utilizing mucus glycans as a nutritional source that can result in inflammatory bowel disease, ulcerative colitis and Crohn’s disease [73]. Because of their potentially adverse consequences, the ability to degrade and utilize mucin places a question mark on the safety of some of the tested strains here [74]. Bacteria associated with fermented foods generally can tolerate biogenic amines, and therefore, such microbes must be checked for the presence of active amino acid decarboxylase before declaring them as safe for human use. These nitrogenous compounds are associated with several adverse health effects, including nausea, rashes, heart palpitations, flushing, headache and hypertension [75,76]. As reported before, kefir-associated bacteria are not significantly bioaminogenic [2], which is corroborated here. Interestingly, these enzymes have been reported in *Enterococcus* species from other fermented products [76,77].

Antimicrobial resistance (AMR) is a significant threat to public health [78]. WHO identifies its prevalence in South Asian countries as especially concerning [79]. Food and food production systems are often the main conduits of AMR spread [80], and fermented foods figure very prominently in this [27]. *Enterococcus* derived from animal sources is frequently associated with transferable antibiotic resistance and virulence [28,41, 81]. This contrasts with the potential of commensal enterococci to act as probiotics [28], prompting global regulators like EFSA to curtail its use in the functional food industry [82].

*Enterococcus* and *Lactococcus* species have become commonplace due to their inherent ability to thrive under challenging conditions [83,84]. The fact that enterococci have acquired resistance to antibiotics over time is a consequence of this inherent ability, which poses a challenge for mass-producing them as commercial probiotics and precludes them from getting GRAS and QPS approvals [85]. In contrast, *Lactococcus* strains are susceptible to several antibiotics but are intrinsically resistant to only a few [86]. This underscores the need to comprehensively map out antibiotic susceptibilities in candidate strains before they are grown on a large scale for commercial use [87]. *Enterococcus* strains of kefir origin are often not ampicillin-susceptible [28], which was also the case in half of the strains tested here. *Enterococcus* strains of dairy origin are usually susceptible to ampicillin [88], which contradicts our findings. Enterococci are intrinsically resistant to low levels of penicillin, but the acquisition of *β*-lactamases and point mutations in genes coding for penicillin-binding regions empowers them to resist high levels of penicillin [89]. Susceptibility to ampicillin in *L. lactis* strains derived from nabeez aligns with previous reports [90], but finding ampicillin resistance in a single strain of *L. lactis*, i.e. NPL1436, is unusual and defies precedence. The variation in resistance patterns of our *L. lactis* strains could be attributable to plasmid acquisition [91]. As seen here, gentamicin resistance has been noted in *Enterococcus* strains of dairy origin [92] and is deemed intrinsic [89]. High-level streptomycin resistance (~1,024 µg ml^−1^) in an *E. durans* strain found here suggests that it could be an acquired trait [89]. Past reports on erythromycin resistance in enterococci derived from kefir [28] and dairy origin [92] support its presence in the enterococcal strains of milk kefir origin characterized here. This question uses erythromycin as a substitute for penicillin in current clinical practice, as the latter has proven ineffective [87]. Tetracycline resistance, as seen in two strains of *E. faecium* here, is widespread among *Enterococcus* [93]. Similarly, tetracycline resistance seen in *L. lactis* strain NPL1436_Nb_ has previously been described in dairy-origin strains of *L. lactis* [84]. The genetic determinants of tetracycline resistance can reside on mobile genetic elements, e.g. plasmids and transposons [84], which constitute a threat of transmission to other bacteria and therefore must be examined at the genome level. Phenotypically demonstrable chloramphenicol resistance, as seen in all of the *E. faecium* strains here, has been attributed to proteins encoded by the *lacA* or *lacG* operon and is part of the mannose phosphotransferse system [88]. Resistance and susceptibility patterns to clinically essential antibiotics vary among strains of the same species [92,94,96] and are a crucial factor in the decision to recommend or reject a candidate strain for commercial application [82,97]. Whether a particular resistance phenotype is intrinsic or extrinsic has a bearing on this decision because there is a history of food-derived microbial strains carrying transmissible antibiotic resistance [92,94].

The strains deemed safe were functionally characterized for their antimicrobial character, as a primary benefit of fermented foods is that they inhibit pathogen populations and neutralize their toxins [98]. Unlike our findings, water kefir-derived micro-organisms can better inhibit bacterial and fungal pathogens because of the strong organic acids they produce [8]. The antibacterial activity of *Enterococcus* strains is a well-established phenomenon [81]. The robust antagonistic ability of dairy-origin *E. durans* is documented, which adds to its potential as a probiotic [41,92]. Our finding that *E. faecium* strains recovered from kefir are strongly inhibitory of human bacterial pathogen species resonates with earlier findings [99]. In the same vein, *L. lactis*, found in Nabeez here, is common in fermented foods and is known to have robust antipathogenic effects [100].

Though *Enterococcus* species have a significant role in fermenting various food matrices, their occurrence in kefir has not received much scientific attention [3]. The fact that significantly more enterococci are found in homemade rather than industrially prepared kefir deserves more attention [3]. Antagonistic strains of *E. durans* have been isolated from milk kefir [101] and Tibetan kefir beverages elsewhere [102]. The prevalence of *E. durans* in kefir has been controversial since some claim that it is a common inhabitant of milk kefir [28,99], which is also corroborated by our findings as well. However, this contrasts with other investigations where the microbial makeup of kefir grains has been shown to consist predominantly of *Lactobacillus* spp. [6]. Numerous investigations have attempted to address whether enterococci are a typical member of its microbiota with mixed results. *E. faecium* is reportedly absent in water kefir [103], contradicting our findings, but enterococci have been found in milk kefir grains [61]. The challenges underlying this issue have been deliberated upon in some investigations [28]. As part of the LAB groups, the lactococci are vitally crucial in fermentation processes [104], but they are relatively infrequent in date palm fruit [105].

No antagonistically significant *Lactococcus* strains were recovered from our kefir samples, contrary to previous reports that suggest their presence in lactococci of kefir provenance [101]. Interestingly, *L. lactis* is more abundant in home-based kefir than in commercial ones [3,6]. The type of milk used for making kefir significantly influences the fermentation product’s microbial composition [54]. Their spatial–physical associations with grain material can significantly challenge their recovery using culture-dependent methods [6].

A good probiotic can withstand and maintain its viability in gut transit. Different challenges a probiotic strain is expected to encounter in its journey through the human gut include digestive enzymes, acids, bile, pancreatic juices, pepsin and competition with other micro-organisms for space and food [28,92]. Therefore, a set of preliminary tests for the probiotic potential and safety assessments has been recommended by FAO/WHO [106] for assessing would-be probiotic candidates. Choosing kefir as a likely source of prospective probionts is a good choice since several studies have reported commensals in kefir that are remarkably attuned to tolerating human gut-associated physiological stressors [69,107]. The salivary lysozyme acts as an antimicrobial by breaking down the bacterial cell wall and represents the first challenge to the survival of an orally administered probiotic. *Enterococcus* strains that claim a fermentation provenance are usually well tolerant of high lysozyme concentrations [108]. However, this was not the case here, which is an aspect that needs more investigation. Acid tolerance represents a strain’s durability in the stomach, whereas bile resistance refers to its metabolic activity in the presence of bile in the intestine [108]. Exposure to strong acid was limited to 3 h reflecting the actual food retention time in the human stomach [28]. Bile inhibits the bacterial cells by denaturing cell membranes [108], and concentrations used for *in vitro* testing simulate those found in the human intestine [109]. Several studies suggest the ability of *Enterococcus* species from different artisanal foods to be remarkably tolerant of human GIT-related stresses [108,110]. *E. durans* strains claiming a kefir provenance are adept at withstanding gastric acidity and intestinal bile [28]. However, our finding of strains of *E. faecium* that are poorly tolerant of GIT stresses negates what has been reported about the species elsewhere [110,111]. However, good tolerance of GIT-associated stresses seen in *L. lactis* NPL1428 is unsurprising since there is a reported precedent for it [112]. Gut bacteria act on dietary proteins and deaminate the aromatic amino acids, resulting in the making of phenolic compounds in the GIT, which are toxic and inhibitory towards probiotic strains [108,112]. Exogenous probiotics must be able to tolerate it to some extent if they are to succeed. The ability to tolerate high doses of phenol (~0.6% w/v), which is the case here, indicates resistance to its bacteriostatic effects [113]. *L. lactis* can typically withstand exposure to high levels of phenolics as corroborated by this study [112]. To assess whether a strain is functionally valuable under human GIT conditions, a well-established *in vitro* static model developed by the INFOGEST international consortium was adopted [51]. It is advantageous because of its simplicity, rapidity and cost [114]. The fact that it can tolerate a plethora of gut-associated stresses makes its probiotic candidature interesting since, in the past, the *Lactococcus* cells were deemed sensitive to SIFs [90]. Strains from fermented products, better tolerators of GIT stresses, have good prospects as novel probiotics.

Even after overcoming the harsh chemical barriers, the most significant obstacles for a probiotic are adherence and colonization. Although adhesion as a screening criterion arguably does not benefit the consumer per se, it indicates a would-be probiont’s ability to compete with enteropathogens for space and food. It helps create a transient host–microbe relationship, allowing more time for the strains to transfer their benefits to the human host [115]. Good autoaggregation capabilities help adhesion and intestinal colonization [94]. The strains of *E. faecium* obtained from kefir and nabeez here had autoaggregation and adhesion potentials, which aligns with a previous finding involving *E. faecium* of dairy provenance [94].

Antioxidant activity helps neutralize cytotoxic free radicals and improve the immune system [25]. Nabeez and kefir are renowned for their antioxidant potentials imparted via their microbial and chemical composition [25,116]. LAB, especially *Lactococcus* species, have a strong history of demonstrating antioxidant potential and increased superoxide dismutase activity [117,118]. Similarly, *E. durans* derived from dairy can scavenge reactive oxygen species [119]. Probiotic candidates with higher antioxidant activity are promising for reducing free radical-related diseases [117]. Higher EPS production in our strains can also be considered a natural antioxidant and antibacterial agent [120]. It is known that EPS produced by bacteria enhances its survival during stress and is also of medical benefit because of its anticancer, antiviral and immune-stimulatory qualities [121]. Other health-promoting qualities demonstrated by the strains investigated here were the substantial cholesterol-absorbing capacities of *E. faecium* and *E. durans* strains, which have previously been reported [94,122]. The cholesterol-lowering benefits of an *L. lactis* strain from an Egyptian fermented beverage validate our findings [123]. Cells of LAB spp. including *L. lactis* cannot only adsorb free cholesterol in their cell walls and lower its concentration in the surroundings [123], but their BSH activity also facilitates cholesterol lowering [124], both of which were noticeably present in the *E. duran*s and *L. lactis* strains which were part of this study. The occurrence of robust BSH activity in food-origin microbes is well-known from investigations elsewhere [94].

Probiotic strains that can metabolize a broader set of carbohydrates are better suited for adapting to diverse habitats [125], can benefit the host’s gut microbiota and alter its composition in favourable ways [126,127]. Metabolic versatility is a hallmark of water kefir strains [20]. The ability of our strains to ferment inulin is worth noting since inulin (fructans) has been recommended as an excellent substrate to increase the grain’s biomass and granule size by supporting microbial growth [90,128].

PCA was preferred for data representation and identifying a probable candidate through dimensionality reduction [129]. In contrast to other selection methods, it considers even slight variations introduced by testing variables, thus giving a better profile of the traits and attributes [130]. Several researchers have utilized this strategy to select the best potential probiotic candidates [129,131]. PCA ranks *L. lactis* strains (NPL1428 and NPL1436) as the most promising probiotic candidates with high GIT resilience and anti-oxidant potential, the latter property having therapeutic implications [132]. Because of the presence of antibiotic resistance in strain NPL1436, we recommend strain NPL1428 as the most appropriate for human probiotic applications.

## Conclusion

Artisanally made fermented beverages, kefir and nabeez, which are part of the global food heritage, remain a relatively untapped source of novel potentially probiotic LAB strains. The *Enterococcus* and *Lactococcus* strains isolated from kefir and nabeez were found to be susceptible to clinically relevant antibiotics and were innocuous. Antagonistic strains derived from kefir were especially active against human-centric bacterial pathogens, which makes them well suited for use in clinical strategies for mitigating communicable disease burdens. Our findings suggest that these strains, particularly *L. lactis* NPL1428, can survive the deleterious effects of oral lysozyme, stomach acidity, small intestine’s bile secretions and gut phenolics. This strain, because of its ability to adhere to mucin, make biofilms and show functional properties that could benefit the human host, makes it a strong candidate for human medicinal use. Further *in vivo* and clinical studies would help determine the probiotic potential use of these kefir-derived LAB strains.

## Abbreviations

AMR: antimicrobial resistance
CFS: cell-free supernatant
DW: distilled water
EFSA: European Food Safety Authority
EPS: exopolysaccharide
FAO/WHO: Food and Agriculture Organization/World Health Organization
GIT: gastrointestinal tract
GRAS: generally recognized as safe
HCA: hierarchical cluster analysis
HRS: hydroxyl radical scavenging
LAB: lactic acid bacteria
MRS: de Man, Rogosa and Sharpe
NPL: National Probiotic Lab
PCA: principal component analysis
QPS: qualified presumption of safety
SAS: superoxide anion scavenging
SGF: simulated gastric fluid
SIF: simulated intestinal fluid
SSF: salivary fluid
T_d_: doubling time
TFFB: traditional fermented foods and beverages
UHT: ultra-high temperature
